# SNP Identification through Transcriptome Analysis of the European Brown Hare (*Lepus europaeus*): Cellular Energetics and Mother’s Curse

**DOI:** 10.1371/journal.pone.0159939

**Published:** 2016-07-26

**Authors:** Grigoris D. Amoutzias, Themistoklis Giannoulis, Katerina A. Moutou, Anna-Maria G. Psarra, Costas Stamatis, Andreas Tsipourlianos, Zissis Mamuris

**Affiliations:** Department of Biochemistry and Biotechnology, University of Thessaly, 41221, Larissa, Greece; University of Thessaly, GREECE

## Abstract

The European brown hare (*Lepus europaeus*, Pallas 1778) is an important small game species in Europe. Due to its size and position in the food chain, as well as its life history, phenotypic variation and the relatively recent speciation events, brown hare plays an important role in the structure of various ecosystems and has emerged as an important species for population management and evolutionary studies. In order to identify informative SNPs for such studies, heart and liver tissues of three samples from the European lineage and a three-sample pool from the Anatolian lineage were subjected to RNA-Sequencing analysis. This effort resulted in 9496 well-assembled protein-coding sequences with close homology to human. After applying very stringent filtering criteria, 66185 polymorphic sites were identified in 7665 genes/cds and 2050 of those polymorphic sites are potentially capable of distinguishing the European from the Anatolian lineage. From these distinguishing mutations we focused on those in genes that are involved in cellular energy production, namely the glycolysis, Krebs cycle and the OXPHOS machinery. A selected set of SNPs was also validated by Sanger sequencing. By simulating the three European individuals as one pool, no substantial informative-SNP identification was lost, making it a cost-efficient approach. To our knowledge this is the first attempt to correlate the differentiation in both nuclear and mitochondrial genome between the two different lineages of *L*. *europaeus* with the observed spatial partitioning of the lineages of the species, proposing a possible mechanism that is maintaining the reproductive isolation of the lineages.

## Introduction

The European brown hare (*Lepus europaeus*, Pallas 1778) is an important small game species in Europe. According to molecular data most of the African and European species of hare studied could have originated during the last 3 Myr, i.e. in the second half of the Pliocene and at the Plio/Pleistocene boundary [1]. Brown hare originated on the open steppe grasslands of Eurasia, adapted very successfully to mixed arable agriculture and was introduced successfully into other countries such as New Zealand and Argentina [2]. Due to its size and position in the food chain, brown hare plays an important role in the structure of various ecosystems. Nevertheless, its populations have decreased dramatically ([3] and literature cited therein).

**Fig 1:**
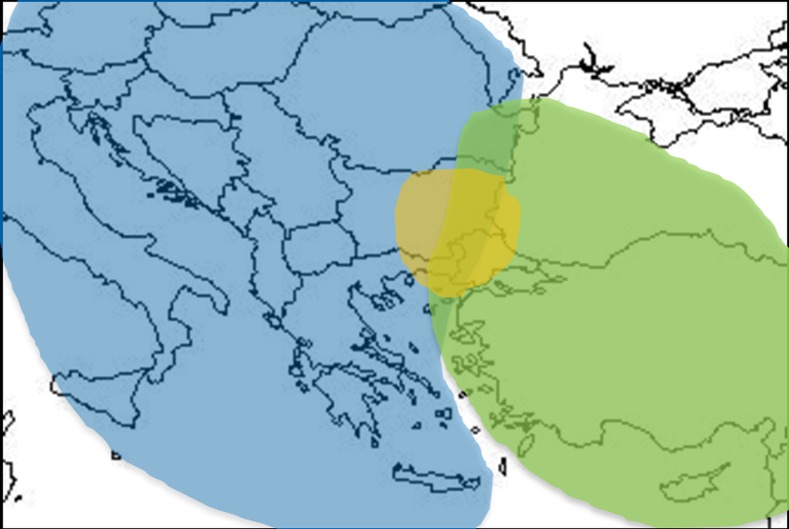
*Lepus europaeus’* distribution and phylogeny. The occurrence of two distinct, well separated, mtDNA lineages, in Europe (blue) and in Anatolia (green) [4]. No European haplotypes have been detected in Anatolia, and no Anatolian haplotypes have been detected in North-Western, Central, South Greece or the rest of Europe. However, there is an introgression zone with the two haplogroups present in Bulgaria and North-Eastern Greece (orange).

A large number of studies over the last 20 years have focused on its distribution, ecology, biology, population dynamics and evolutionary history and have helped us to better understand fundamental issues concerning mammalian micro- and macro-evolution, conservation biology and local adaptations. Data that were based on nuclear DNA markers have supported relatively high rates of gene flow across large geographical ranges, whereas mitochondrial DNA (mtDNA) analysis has revealed a higher degree of spatial partitioning [4, 5]. A comprehensive phylogeographic analysis, based on mtDNA variability of >1200 individuals from Europe, Asia Minor, Middle East and Cyprus identified three major haplogroups with a clear phylogeographic signal that reflected the presence of late-Pleistocene refugia in the central⁄southern Balkans and in Anatolia ([4]; unpublished data). The latter study identified two distinct mtDNA lineages, one in Europe and one in Anatolia that were well separated, whereas the European lineage was further subdivided in a Greek and a Central European one (Fig 1) [4]. So far, no European mtDNA haplotypes have been detected in Turkey and Israel. Also, no Anatolian mtDNA haplotypes have been detected in North-Western, Central, South Greece or the rest of Europe. Similarly, Greek mtDNA lineages have not been detected in north-central Europe. However, there is a large introgression zone with all haplogroups present in Bulgaria and North-Eastern Greece (Fig 1). The population dynamics and the spatial partitioning of the introgression zone have been well described in a study of Antoniou and her colleagues [6] by using mitochondrial sequences of the control region along with microsatellite data for 10 loci. The existence of two diversified clades of mtDNA was confirmed once again, along with the partitioning of the samples in five major groups, the parental populations that mate and produce offspring and three groups of hybrids (F1, F2 hybrids and backcrosses with parental populations). Other studies also support the differentiation in nuclear level, using functional genes related to immune response [7] as well as coding and non-coding regions of the Y chromosome [8]. To summarize, in the above studies, a broad area of sampling have been used and the existence of the two lineages has been well supported so far from mitochondrial and nuclear data.

A big decline in the number of individuals of the species caused by extensive hunting and spread of diseases raised the need for restocking operations in many European countries [1, 4, 9,10]. Although various restocking operations could be partly responsible for the presence of unexpected haplotypes in certain areas, previous studies traced a strong phylogeographic signal throughout all the regions under study and especially between Europe and Anatolia [1, 4, 9,10]. This distribution pattern observed between populations within the species may reflect micro-evolutionary processes that have resulted in local adaptations of specific gene pools.

Adaptive mechanisms and their genetic basis are nowadays in the centre of evolutionary biology and molecular ecology studies. Most of the studies use neutral markers to estimate the differentiation and genetic variability of natural populations, to elucidate evolutionary histories and to deduce the influence of humans and the environment on demographic parameters and gene flow. However, neutral loci cannot provide information on the mechanisms shaping adaptive variation, the relative contribution of different micro-evolutionary processes and the action of natural selection in the retention of adaptive polymorphisms [11]. So far, mtDNA was considered neutral in selection terms [12]. However, since mtDNA encodes for proteins that participate in the oxidative phosphorylation (OXPHOS), it may affect fitness by altering metabolic performance [13]. OXPHOS is a great paradigm of genome cooperation: mtDNA- and nuclear- encoded subunits form the five complexes of the machinery that produces ATP via electron transport. Mitochondrial DNA mutation rates are high and new mtDNA alleles are being continuously generated [12]. These alleles persist, even if they are slightly deleterious, because the lack of recombination in mtDNA leads to an inevitable accumulation of linkage disequilibrium. The higher mutation rates of mtDNA calls for tight co-evolution of the cooperating nuclear genes. In several instances this cooperation may break affecting the performance of the machinery. In the study of Smith et al [14] it was found that brown hares carrying different haplotypes of mtDNA exhibited impaired reproductive success when crossing with each other in captivity, proposing the phenomenon of mother’s curse: mtDNA mutations that affect OXPHOS efficiency have higher impact on sperm cells due to its high energy demands, thus reducing male fertility, unlike the ovum that displays low energy demands. Through such mechanism, mtDNA mutations may act as drivers of adaptive evolution in nuclear genes.

The aim of this study is to analyze the trancriptome polymorphism, using transcriptome shotgun sequencing (RNA-seq), along with mtDNA polymorphism of the European brown hare and investigate sequence variation from individuals that belong to the two distinct major mtDNA phylogroups, the European and the Anatolian one. The purpose was (a) to elucidate the relations between the energy production procedures and the local adaptations of populations of the species and (b) to provide an explanation for the absence of “Anatolian” haplotypes in Europe and vice-versa despite the presence of the large introgression zone bearing all haplogroups in Bulgaria and North-Eastern Greece for more than 10000 years. A plausible hypothesis could be the reduced fitness of hybrids due to impaired efficiency in energy production. The study focused on genes involved in cellular energetics, namely, the glycolysis, Krebs cycle and the OXPHOS machinery. The first two are governed solely by nuclear-encoded genes whereas the later demands the efficient cross-talk of nuclear- and mitochondrial-encoded genes.

## Materials and Methods

### Ethics statement

The hare samples were collected opportunistically (no active capture, killing and sampling of wild animals specifically for this study was performed) from animals hunter-harvested by members of Greek Hunting Federation of Macedonia and Thrace and Cyprus (Licenced by the Greek Ministry of Rural Development and Food and the Cypriot Service of Game and Fauna respectively), from species considered quarry and during the hunting seasons, according to the prerequisites of the Greek Legislation (FEK3100 Β’/06-12-2013) or the Cypriot Legislation (ΕΕΚD 4617 8/8/2014), thus special approval was not necessary and steps to ameliorate suffering were not applicable in this study.

### Sample collection, RNA/DNA extraction and sequencing

**Fig 2:**
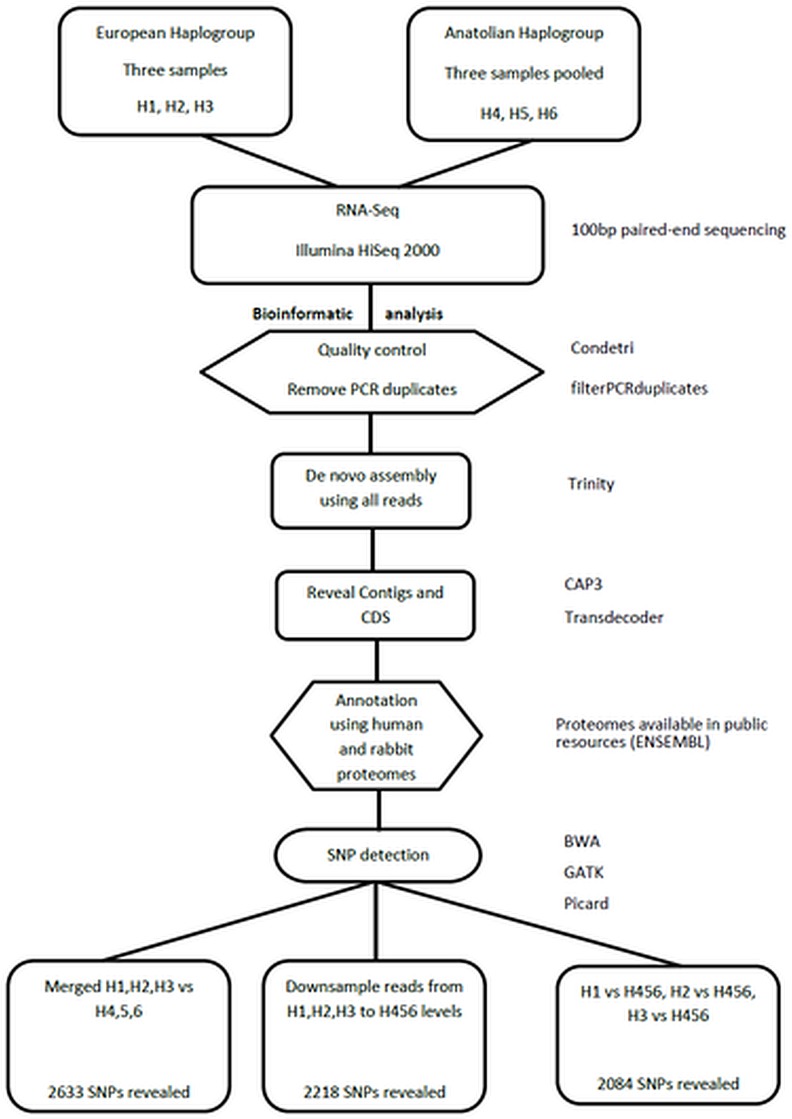
RNA-seq analysis pipeline. Schematic representation of the bioinformatics pipeline used in RNA-Seq analysis and SNP detection in nuclear-encoded genes.

For the RNA-Seq analysis, total RNA was extracted from the heart and liver of six (6) specimens of *Lepus europaeus*, using TRI Reagent (Sigma-Aldrich, Product No.T9424) according to manufacturer’s protocol. For each individual, RNA from both tissues was pooled together. Three of the samples belonged to the European lineage (originating from North Eastern Greece), whereas the other three belonged to the Anatolian haplogroup (all three from Cyprus). The tissues from each individual were immediately placed in separate tubes and held in dry ice until the RNA extraction. Subsequently, the three Anatolian samples were pooled together as one sample (thus reducing their sequencing cost to 1/3), indexed H456, whereas the three European samples, indexed H1, H2 and H3, were processed separately (Fig 2).

The four RNA samples were dispatched to Macrogen Inc. (Korea) for analysis. Poly-A containing RNA molecules were purified and fractionated. The RNA fragments were used for cDNA synthesis, library construction and were sequenced on an Illumina HiSeq 2000, providing 100 nt long Paired-End reads. The filtered fastq files were submitted to the NCBI Sequence read archive with accession numbers SAMN03382608-SAMN03382611.

For mtDNA analysis DNA extraction was performed in the six samples using PureLink Genomic DNA kits (Invitrogen, Carlsbad, CA) according to manufacturer’s protocol, followed by mtDNA isolation using PureLink® Genomic DNA Mini Kit according to manufacturer’s protocol. The mtDNA samples were also dispatched to Macrogen Inc (Korea) and were sequenced on an Illumina HiSeq 2000, providing 100 nt long PE reads.

### Bioinformatics analyses

#### Assembly step

**Fig 3:**
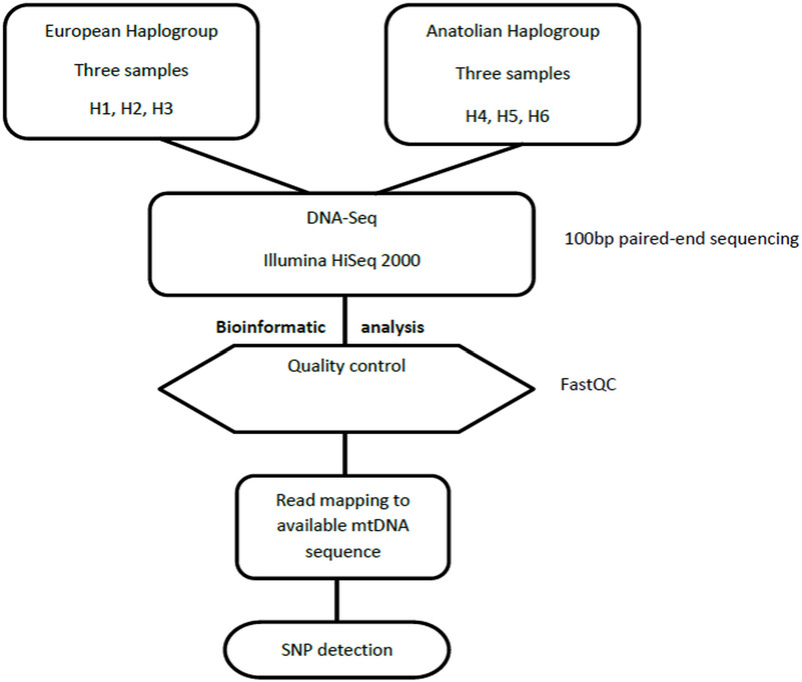
DNA-Seq analysis pipeline. Schematic representation of the bioinformatics pipeline used in DNAseq analysis and SNP detection in mtDNA-encoded genes.

Quality control of sequenced reads was performed with the FASTQC software (http://www.bioinformatics.babraham.ac.uk/projects/fastqc/). Sequence reads were trimmed based on q-score base qualities and filtered for PCR duplicates with the Condetri and the filterPCRduplicates software [15]. All four samples (H1, H2, H3 –European haplogroup; H456 –Anatolian haplogroup) were pooled into one (H123456) to increase read overlaps and thus improve the assembly of transcripts with the Trinity software (default parameters; [16]). Next, these transcripts were further merged into contigs whenever possible with the CAP3 software (default parameters; [17]). The Transdecoder program, within the Trinity software was implemented (default parameters) to predict the protein coding sequences (cds) from the transcripts (http://transdecoder.github.io/) (Fig 2). For mtDNA sequences, we used the same criteria for quality control and trimming and the reads were mapped on the available mtDNA sequence of *L*. *europaeus* (Accession number NC_004028.1) (Fig 3).

#### Homology search and annotation of nuclear genes

The human (GRC37.71.all.peps) and rabbit (Oryctolagus2.73.all.peps) proteomes were downloaded from ENSEMBL and were used for identifying homologous sequences in the brown hare proteins [18, 19]. Thus, two separate Blastp runs [20] were implemented with an E-value cutoff of 1e-10, where the hare proteins were used as queries and the human and rabbit proteomes as databases respectively. For every hare protein we retained the best blastp hit (based on blast bit score) against human or rabbit proteins. Next, if two or more hare proteins had the same human gene as their closest homolog, we would only retain that hare protein with the longest cds. Successively, we only retained those hare proteins that had good blastp query and subject coverage (≥70%) against their best human and rabbit homologs. The blast results were integrated with annotation from ENSEMBL, concerning ENSEMBL gene id, protein id and function. This integration was done with the ENSEMBL Biomart tool and custom Perl scripts (Fig 2).

#### Mitochondrial sequences

Using the blastn program with default parameters, with the available mtDNA-encoded genes as query, we were able to retrieve the full length of the thirteen protein coding genes of the mtDNA for each of the six samples. The absence of insertions, deletions or in-frame stop codons within the sequences indicate that they correspond to functional mitochondrial genes and are not derived from pseudogenes from nuclear DNA’s residues that could be present in the sample (Accession numbers KU250057-KU250134).

#### SNP identification

The well assembled protein-coding nucleotide sequences were used as a reference set, upon which BWA aligned the sequence reads of each sample, in order to detect polymorphic sites [21]. Manipulation, filtering, and realignment of polymorphic sites were implemented with the GATK [22] and Picard software tools (http://broadinstitute.github.io/picard/) (Fig 2).

Unified genotyper of the GATK software was run three (3) times (Fig 2), using as cutoff a QVALUE of 30.

- In the first run, the three European samples (H1, H2, H3) were merged together into a pooled one sample (H123) and analyzed against the Anatolian pooled sample (H456). The two pools were treated as hexaploids. The results of this run are saved as a vcf file in supplementary material (S3 File).
- In the second one, the three European samples (H1, H2, H3) were stochastically downsampled with Picard (in a balanced way) and then were merged together into a pooled one, thus generating a new pooled sample (H123_downsampled) of the same number of reads as the H456 pooled sample. Afterwards, the two pooled samples (H123_downsampled and H456) were analyzed against each other and were treated as hexaploids. The results of this run are saved as a vcf file available upon request.
- In the third run, each of the three individual samples (H1, H2, H3) and the 4^th^ pooled sample (H456) were analyzed individually against each other. The results of this run are saved as a vcf file available upon request.

#### Filtering of polymorphic sites

The identified, confident polymorphic sites that could potentially distinguish the European from the Anatolian lineages were filtered by applying five stringent criteria in our analysis. The first criterion was that the alleles observed in one population were not present at all in the other population. The second criterion was that any genotypes observed were supported by a QVALUE of 100 or more. The third criterion was that any allele observed in a certain population was supported by at least thirty sequence reads. The fourth criterion was that in each sample/population we allowed only one read to support an allele not suggested from UnifiedGenotyper in GATK. The fifth criterion was to exclude polymorphic sites from any sequences for which there was evidence that they could be merged close duplicates.

#### Genes involved in Cellular Energetics

Following the annotation and SNP identification, the SNPs of the coding sequences of genes involved in cellular energetics were retrieved and compared among the two haplogroups. The complete list of genes is shown in Table 1. The analysis was made treating the samples as hexaploids to detect their genotypes. A 50%-differentiation criterion was used to make the call for the differentiating SNPs (e.g. genotypes 0/0/0/0/0/0 and 0/0/0/1/1/1, where 0 and 1 are referred to different alleles, are considered differentiated).

**Table 1:** The list of genes involved in cellular energetics that were targeted in the current analysis. Genes involved in Oxidative Phosphorylation are also separated by Complex (Complexes I-V). Genes encoded by the mtDNA are presented as underlined.

Glycolysis	Krebs cycle	Complex I nuclear genes	Complex I mtDNA genes	Complex II nuclear genes	Complex III nuclear genes	Complex III mtDNA genes	Complex IV nuclear genes	Complex IV mtDNA genes	Complex V nuclear genes	Complex V mtDNA genes
HK1	ACO2	NDUFV1	ND1	SDHA	CYC1	CYTB	COX4I1	COXI	ATP5A1	ATP6
GPI	CS	NDUFV2	ND2	SDHB	UQCRC1		COX5B	COXII	ATP5B	ATP8
PFKL	FH	NDUFS1	ND3	SDHC	UQCRC2		COX6A2	COXIII	ATP5C1	
ALDOA	IDH2	NDUFS2	ND4	SDHD	UQCRFS1		COX6B1		ATP5D	
TPI1	MDH1	NDUFS3	ND4L		UQCRH		COX6C		ATP5F1	
GAPDH	OGDH	NDUFS7	ND5		UQCRQ		COX7A2		ATP5G1	
PGK1	SDHA	NDUFS8	ND6						ATP5G2	
PGAM1	SUCLA2	NDUFV3							ATP5H	
PKM		NDUFS4							ATP5J2	
ENO1		NDUFS5							ATP5L	
		NDUFS6							ATP5O	
		NDUFA1							ATP5S	
		NDUFA2								
		NDUFA3								
		NDUFA4								
		NDUFA5								
		NDUFA6								
		NDUFA7								
		NDUFA8								
		NDUFA9								
		NDUFA11								
		NDUFA12								
		NDUFA13								
		NDUFAB1								
		NDUFB1								
		NDUFB2								
		NDUFB3								
		NDUFB4								
		NDUFB5								
		NDUFB6								
		NDUFB7								
		NDUFB8								
		NDUFB9								
		NDUFB10								
		NDUFB11								
		NDUFC1								
		NDUFC2								

### SNP Validation

A selected set of RNA-Seq determined SNPs was validated with Sanger sequencing. The selected SNPs were from the total SNP list and weren’t extracted from the SNPs that could potentially distinguish the two lineages (see Results section below). DNA was extracted from 10 European and 10 Anatolian samples that were not included in the RNA-Seq analysis. The European samples were from Greece (4), Germany (3) and France (3) whereas the Anatolian samples were from Cyprus (5) and Turkey (5). DNA extraction was performed using PureLink Genomic DNA kits (Invitrogen, Carlsbad, CA) according to manufacturer’s protocol. PCR Primers were designed for 54 RNA-Seq detected polymorphic sites (non-synonymous mutations) within 24 genes for validation with Sanger sequencing. The primer sets were designed using the NCBI Primer-Blast algorithm (http://www.ncbi.nlm.nih.gov/tools/primer-blast/) (see S2 Table; worksheet: Primers_Sanger_validation). The cycling conditions consisted of an initial denaturation at 95°C for 5 min followed by 35 cycles of denaturation at 95°C for 30 sec, annealing at specific temperatures for each primer set and extension at 72°C for 30 sec, with a final extension at 72°C for 10min. PCR products were purified using QIAquick PCR Purification Kit (QIAGEN, Hilden, Germany) and sequenced bi-directionally. Sanger sequencing was performed at Macrogen Inc. (The Netherlands). Nucleotide sequences were aligned using ClustalX [23].

## Results

### Identification of homologous sequences in Human and Rabbit

RNA-Sequencing of the three European RNA-Seq samples and of the one pooled Anatolian sample generated between 10 and 17 million paired-end reads for each sample (H1: 10,378,247; H2: 17,869,153; H3: 16,463,647; H456: 16,288,904), after the filtering applied. The Trinity assembly resulted in 174,942 contigs, whereas the extra CAP3 assembly step reduced the contigs to 162,519 of which 147,818 were protein-coding, according to Transdecoder.

After Blastp and the relevant stringent filtering steps mentioned in Materials and Methods, we identified 9496 hare cds/proteins that were the closest and best homologs against human genes (see S1 and S2 Files). Thus, we did a 1:1 mapping between hare and human protein coding sequences. For these hare cds/proteins, the average identity and similarity against human proteins was 89.9% and 93.9% respectively. Also, on average, 95% of each hare cds/protein was aligned with its human homolog in a blastp pairwise alignment, an indication that for this set of 9496 sequences the Trinity and CAP3 assemblies were very good (see S1 Table).

The same steps as above were performed between hare and rabbit sequences, but 8264 hare sequences were retained this time, instead of the 9496 when using the human genes/proteins. This is an expected indication that the human genome is more mature/complete than the rabbit genome. Of note, those 8264 hare sequences had an average 96.6% identity and 97.6% similarity against rabbit proteins. Also, on average, 95% of each hare cds/protein was aligned with its rabbit homolog in a blastp pairwise alignment.

In order to estimate the divergence at the nucleotide level of protein coding sequences between the two closely related species *L*. *europeus* and *L*. *granatensis*, sequences of the latter were obtained from an RNA-seq experiment by Cahais et al [24]. The 9496 cds of *L*. *europeus* were blasted (blastn—evalue cutoff 1e-10) against the 45151 contigs of *L*. *granatensis* and the average nucleotide identity was estimated at 99.29%.

### Identification of SNPs

From the first run (H123 vs H456), and after filtering the vcf file (with a qvalue≥100 and DP≥30 and MQ0 = 0 and MQ≥30 or more), we identified 69,608 polymorphic sites in 7,755 genes/cds. After filtering out the 90 cds that could be merged gene duplicates our list was reduced to 7665 genes/cds that had 66,185 polymorphic sites (see S2 Table; worksheet: 69608_polymorphic_sites & worksheet:7755_genes-cds).

The application of stringent criteria (see Materials and Methods) resulted in 2050 polymorphic sites (2046 SNPs and 4 indels) in 1147 sequences that could potentially differentiate between the two lineages (see S2 Table; worksheet: differentiating_SNPs_2popul). The top ten polymorphic genes of this type had between 9–31 polymorphic sites each. The above filters create two biases, one in favour of highly expressed genes and the other in favour of homozygous sites. Nevertheless, these biases are not our primary concern, because the goal of this specific analysis is to generate a set of polymorphic sites that can distinguish one lineage from the other.

### The effect of pooling the samples of one lineages

Treating the European samples either as individuals or as a pool had the following effects: 2541/2633 confident polymorphic sites (that could potentially differentiate one lineage from the other) would be identified correctly if we investigated each individual separately. This is because in some cases one of the three individuals had a low number of sequence reads for a particular site, whereas another individual of the same population had a high number of reads for the same site. When these samples were pooled together, this difference was ameliorated. In addition, if we applied an extra filter of 10 reads at least for each allele in each individual, then, the number of confidently identified polymorphic sites that are capable of separating the two lineage would drop to 2084/2633 (79%). On top of that, if we applied the additional filter of removing polymorphisms from sequences that are suspected to be merged closely related gene duplicates, the number of polymorphic sites that may separate one lineage from the other drops to 2050. Therefore, the effect of pooling is not as dramatic, when considering the reduction of cost.

### Polymorphic sites in genes involved in cellular energetics

After the assembly/annotation/SNP identification steps, the coding sequences from the genes of interest were retrieved. For this purpose, the dataset of SNPs that derived from the first run of variant calling mentioned above was used, when all the samples of one population were merged in one pool and were compared as hexaploids (H123 vs H456). SNPs belonging to these genes, along with SNPs from other genes with functions related with mitochondrion, were further validated with Sanger sequencing. The complete list of SNPs is provided in Table 2.

**Table 2:** RNA-Seq identified SNPs that were further validated by Sanger sequencing in other individuals of the two lineages. Shaded boxes correspond to SNPs that Sanger sequencing failed to validate.

Gene name	SNP Position	Reference allele	Alternative allele	European	Anatolian Haplogroup	*European*	*Anatolian*
		RNA-Seq	RNA-Seq	Haplogroup	RNA-Seq	*Haplogroup*	*Haprogroup*
				RNA-Seq		*Sanger*	*Sanger*
**UQCRC2**	**1218**	**C**	**T**	**C**	**C/T**	***C***	***C***
**UQCRC2**	**1234**	**G**	**A**	**G**	**G/A**	***G***	***G***
**UQCRC2**	**1263**	**C**	**T**	**C**	**C/T**	***C***	***C***
**NDUFB5**	**54**	**G**	**A**	**G**	**G/A**	***G***	***G***
**NDUFB5**	**55**	**C**	**T**	**C**	**T**	***C***	***C***
**NDUFB5**	**56**	**G**	**C**	**G**	**C**	***G***	***G***
**NDUFB5**	**91**	**G**	**A**	**G**	**A**	***G***	***G***
**ATP5G1**	**178**	**T**	**A**	**A/T**	**T**	***A/T***	***T***
**ATP5H**	**556**	**A**	**C/G**	**A/G**	**A/C**	***A***	***A***
**NDUFA4**	**218**	**G**	**A**	**G**	**G/A**	***G***	***G***
**NDUFS2**	**70**	**G**	**T**	**G/T**	**G**	***G***	***G***
**EPRS**	**1258**	**A**	**G**	**G/A**	**G**	***G/A***	***G***
**RARS**	**242**	**G**	**A**	**G/A**	**G**	***G***	***G***
**RARS**	**280**	**C**	**T**	**C/T**	**C**	***C***	***C***
**RARS**	**312**	**A**	**G**	**A/G**	**A**	***A/G***	***G***
**DARS2**	**334**	**G**	**A**	**G**	**G/A**	***G***	***A***
**DARS2**	**554**	**A**	**C**	**A**	**A/C**	***A/C***	***A/C***
**DARS2**	**1405**	**G**	**A**	**G**	**G/A**	***G***	***A***
**DARS2**	**1600**	**A**	**G**	**A**	**A/G**	***A***	***A***
**ATP5J2**	**275**	**G**	**A**	**G/A**	**G**	***G/A***	***G/A***
**ATP5J2**	**465**	**C**	**A**	**C/A**	**A**	***C/A***	***A***
**ATP5J2**	**474**	**T**	**C**	**T/C**	**T**	***T/C***	***T/C***
**ETFA**	**139**	**G**	**C**	**G**	**G/C**	***G/C***	***G/C***
**NDUFAF7**	**1145**	**A**	**C**	**A/C**	**A/C**	***A/C***	***A***
**NDUFAF7**	**1215**	**G**	**T**	**G**	**G/T**	***G***	***G***
**NDUFAF7**	**1248**	**G**	**A**	**G/A**	**G/A**	***G/A***	***G***
**NDUFAF1**	**32**	**C**	**T**	**C/T**	**C**	***C/T***	***C***
**NDUFAF1**	**67**	**C**	**G**	**C/G**	**C**	***C/G***	***C***
**NDUFAF1**	**91**	**C**	**T**	**C/T**	**C**	***C***	***C***
**NDUFB6**	**386**	**A**	**G**	**A/G**	**A**	***A***	***A***
**NDUFV2**	**519**	**A**	**G**	**A/G**	**A/G**	***A/G***	***A***
**OXA1L**	**111**	**G**	**A**	**G/A**	**G**	***G/A***	***G***
**OXA1L**	**122**	**G**	**A**	**G/A**	**G**	***G/A***	***G/A***
**OXA1L**	**145**	**G**	**C**	**G**	**G/C**	***G***	***G***
**SCO1**	**409**	**G**	**A**	**G/A**	**G**	***G***	***G***
**SCO1**	**800**	**A**	**T**	**A/T**	**A**	***A***	***A***
**SCO1**	**804**	**C**	**T**	**C**	**C/T**	***C***	***C***
**SDHD**	**91**	**G**	**A**	**G/A**	**G**	***T/G***	***G***
**SDHD**	**93**	**C**	**T**	**C/T**	**C**	***C/T***	***C***
**SDHD**	**157**	**C**	**T**	**C/T**	**C**	***A/C/T***	***C/T***
**TFAM**	**315**	**A**	**T**	**A**	**A/T**	***A***	***A***
**TFAM**	**371**	**G**	**C**	**G/C**	**G/C**	***G/C***	***G***
**SARS2**	**487**	**T**	**C**	**T/C**	**C**	***T/C***	***C***
**FARS2**	**140**	**G**	**A**	**G/A**	**G**	***G***	***G***
**FARS2**	**663**	**G**	**C**	**G/C**	**G**	***G/C***	***G***
**FARS2**	**680**	**A**	**G**	**A**	**A/G**	***A***	***A***
**FARS2**	**693**	**G**	**A**	**A/G**	**A/G**	***A/G***	***G***
**FARS2**	**708**	**T**	**C**	**T/C**	**T/C**	***T/C***	***T***
**FARS2**	**756**	**A**	**G**	**A/G**	**A/G**	***G***	***A***
**LARS2**	**2434**	**G**	**A**	**G**	**G/A**	***G***	***G***
**LARS2**	**2489**	**T**	**C**	**T/C**	**C**	***T/C***	***C***
**LARS2**	**2509**	**G**	**C**	**G**	**G/C**	***G***	***G***
**LARS**	**1085**	**T**	**C**	**T/C**	**T**	***C***	***T***
**COX5B**	**356**	**A**	**G**	**A/G**	**A/G**	***A***	***G***

Subsequently, the SNPs in these genes for each separated lineage were identified and compared in order to detect differences between the lineages/haplotypes. This procedure was run separately for each step of energy production: the glycolysis, the Krebs cycle and the oxidative phosphorylation (OXPHOS). The rate of total mutations per nucleotide and the rate of non-synonymous mutations per nucleotide differentiating the European from the Anatolian lineage was calculated (Table 3). Interestingly, the OXPHOS nuclear genes exhibited the same level of total differentiating mutations with the glycolysis and the Krebs cycle, whereas non-synonymous mutations in the nuclear genes of the OXPHOS were 3.5- and 2.7-fold more than in the glycolysis and the Krebs cycle, respectively. The thirteen mtDNA-encoded genes exhibited a 4.6- and 3.5-fold higher rate of non-synonymous mutations compared with the glycolysis and the Krebs cycle.

**Table 3:** The rate of total and non-synonymous differentiating mutations, respectively, for the three energy producing procedures. In the OXPHOS, rates are also presented for each of the five complexes separately, distinguishing between nuclear and mtDNA-encoded genes.

Energy Production Procedure		Mutations/nucleotide	Non-synonymous Mutations/nucleotide
*Glycolysis*		0.004685	0.000344
*Krebs cycle*		0.004060	0.000451
*OXPHOS*		0.008056	0.001258
*OXPHOS*	*nuclear*	0.003691	0.001212
	*mtDNA*	0.022641	0.001409
*Complex I*	*nuclear*	0.003900	0.001282
	*mtDNA*	0.020899	0.001583
*Complex II*	*nuclear*	0.005405	0.000257
*Complex III*	*nuclear*	0.003373	0.000930
	*mtDNA*	0.033333	0.001754
*Complex IV*	*nuclear*	0.005391	0.003732
	*mtDNA*	0.016943	0.000664
*Complex V*	*nuclear*	0.001978	0.000923
	*mtDNA*	0.040677	0.002259

While the glycolysis, the Krebs cycle and the complex II are encoded solely by nuclear genes, the complexes I, III, IV and IV of OXPHOS require mitochondrial and nuclear encoded genes for their formation. For this reason, we calculated the rate of total mutations per nucleotide and the rate of non-synonymous mutations per nucleotide for the mtDNA and nuclear encoded-genes, respectively, for each of the five (5) complexes (I-V) of OXPHOS, to allow the comparison on equal grounds. Complex II, which is encoded solely by nuclear genes, had approximately the same rate of accumulating differentiating mutations with the glycolysis and the Krebs cycle, yet a lower rate of non-synonumous mutations. The nuclear subunits of Complex IV, although they accumulate differentiating mutations at a same rate with the glycolysis and the Krebs cycle, they exhibited a 10- and 8-fold higher rate of non-synonumous mutations than the glycolysis and the Krebs cycle, respectively. Generally, nuclear subunits of OXPHOS showed a little difference in the rate of total mutations compared with the glycolysis and the Krebs cycle, but when the non-synonymous rates were compared, the differentiation was at least 2-fold higher in the OXPHOS genes. MtDNA-encoded subunits had higher rates in general in accumulating mutations differentiating the two lineages, almost five times higher than the glycolysis and the Krebs cycle, whereas they accumulated non-synonymous mutations at a lower rate.

### COI Barcodes

In order to explore further the differentiation between the two lineages, we used the COI barcoding system proposed by Hebert and his colleagues [25], when a proportion of the mtDNA-encoded gene of COI is used to identify even close related species. For this purpose, we used our data combined with data available from BOLDSystems (http://www.barcodeoflife.org/) for all the *Lepus* species available. The species selected were: *Lepus americanus*, *L*. *capensis*, *L*. *comus*, *L*. *coreanus*, *L*. *granatensis*, *L*. *hainanus*, *L*. *mandschuricus*, *L*. *microtis*, *L*. *oiostolus*, *L*. *peguensis*, *L*. *sinensis*, *L*. *timidus*, *L*. *tolai*, *L*. *townsendii*, *L*. *americanus and the sequences we derived in this analysis for L*. *europaeus*, where we treated each lineage as a separate group. The average differentiation among species was 6.77%, while the differentiation between the European and the Anatolian lineage was found 1.74%. Interestingly, there were species pairs which showed lower differentiation than the two lineages. These pairs were: *L*. *granatensis vs L*. *coreanus*, *L*. *arcticus vs L*. *granatensis and L*. *coreanus*, *L*. *timidus vs L*. *granatensis*, *L*. *coreanus and L*. *arcticus*, which had a distance between 0.31 and 1.11%.

## Discussion

This genetic break at the margin between Anatolia and the surrounding areas has been observed in a variety of species, the dispersal of which includes these specific territories [26]. Some paradigms include the European green toad [27], the long-fingered bat [28], the yellow-necked fieldmouse [29] and the European grasshopper [30]. In the majority of these cases, mitochondrial DNA markers have been used in order to reveal the differentiation between the geographically isolated populations of the different species.Although mitochondrial DNA markers have proven their usage as molecular markers for inferring phylogenies, population dynamics and evolutionary patterns, they tend to show some disadvantages when they are used self-standing. Mitochondrial DNA, due to the uniparental inheritance and the lack of recombination is vulnerable to phenomena of selective sweeps, which tend to reduce the polymorphism in species level. Also the uniparental inheritance creates a bias in which the marker does not reflect the history of the species as a whole but only that of the female portion. For example, the existence of the hybridization zone in North Greek territory and Bulgaria [antoniou] as well as the hybridization between the *L*. *europaeus and L*. *timidus* in Switzerland, Scandinavia and Russia, [5,31,32] were revealed by application of mtDNA markers and were confirmed using nuclear markers. Moreover, the use of mtDNA as marker for DNA barcoding relies on the low levels of variation within a species compared with intraspecific variation and monophyly of mtDNA within species. Case studies have made clear that this general pattern may not be true for all species. In a review of Funk and Omland [33], using mtDNA data, they suggested that 23% of species examined may not be monophyletic for mtDNA sequences. The disadvantages of the application of solely mtDNA markers discussed above underline the need for combined studies of mitochondrial and nuclear markers in order to infer more accurate theories about species phylogeny and evolutionary processes currently acting on populations. However, in *Lepus europaeus* the differentiation between Anatolian and European lineages has been supported so far by population studies using one or a small number of mitochondrial genes [4, 34], functional regions of the nuclear genome related to immune response [7] and a combination of coding and non-coding regions of Y chromosome [8].

This is the first study to use genome-scale sequence data in order to investigate the level of differentiation and find a possible cause of the lack of the Anatolian lineage in Europe and vice versa, despite the presence of all haplogroups in North-Eastern Greece and Bulgaria. Except of the genomes of two lagomorph species, of the American pika (*Ochotona princeps*) and of the European rabbit (*Oryctolagus cuniculus*), that have been sequenced and assembled by the Broad Institute in the framework of the Mammalian Genome Project [18], to our knowledge this is the first attempt to obtain transcriptome sequences by RNA-Seq in *L*. *europaeus* and the second, after the iberian hare *L*. *granatensis* [24, 35], within the genus *Lepus* that is comprised of 32 species with worldwide distribution [36].

RNA-Seq (heart and liver tissues) of samples from the European and Anatolian phylogenetic lineages resulted in 9496 well-assembled protein coding sequences. After applying very stringent filtering criteria, 66185 polymorphic sites were identified in 7665 genes/cds. The above numbers do not reflect the real level of polymorphism, because they are based on RNA-Seq data, where the sequencing coverage is not uniform among the various genes. Therefore, for highly covered/expressed genes, the current estimated (by RNA-Seq) level of polymorphism is closer to the true one, whereas for lowly covered/expressed genes the level of polymorphism is most probably underestimated.

Fifty four of those sites situated in 24 genes were validated with Sanger sequencing in 10 individuals from each lineage. In addition, 2050 of those polymorphic sites (found in 1147 genes) are potentially capable of distinguishing the members of one lineage from the other. Although, for this analysis, three individuals per lineage were used, the strictness of the criteria can ensure the validity of the SNPs that were uncovered from the analysis. However, the actual number of lineage-separating polymorphisms is bound to be lower.

An interesting finding in energy production processes, after the analysis of these large scale data, was that the glycolysis and the Krebs cycle, governed solely by nuclear-encoded genes, showed a slower rate (almost half) of accumulating differentiating mutations compared with the OXPHOS machinery, which requires the cooperation of mitochondrial and nuclear genome. Moreover, this difference is even higher when comparing the rate of non-synonymous mutations between these three procedures; OXPHOS machinery showed a ~3-fold higher on average rate of differentiating non-synonymous (ns) mutations compared with the glycolysis and the Krebs cycle.

Also, when comparing the rates of nuclear-encoded genes of OXPHOS to the glycolysis and the Krebs cycle, whilst the rate of differentiating mutations was approximately the same, when comparing non-synonymous rates the OXPHOS’ nuclear subunits showed a three times higher rate on average compared with the other two, and the mtDNA encoded genes show a 3.5-fold higher rate compared with the glycolysis and the Krebs cycle.

More interestingly, when the differentiation in every complex of the OXPHOS was computed separately (I-V), the results were more definite: complex II, which is encoded only by nuclear genes, had a similar rate of ns mutations with glycolysis and Krebs cycle while complexes I,III,IV and V have a ~3.5-fold higher rate on average. The bigger divergence was found in Complex IV, where the mitochondrial subunits have the catalytic functions and are showing the lower differentiation among the mitochondrial genes and the nuclear encoded subunits, which have role in assembly and regulation of the activity [37], are showing a nine-fold higher rate on average when compared to glycolysis and Krebs cycle. The role of cytochrome C oxidase (Complex IV) has been found to be responsible in hybrid breakdown in marine copepods [38], where COX activity was significantly reduced in hybrids of crosses between different geographical populations. A similar mtDNA introgression study in *Drosophila* showed a more pronounced COX disruption effect in interspecific versus intraspecific backcrosses [39].

It is very likely that the different rate of adaptive evolution of nuclear genes OXPHOS, as opposed to the other two groups of genes (glycolysis and Krebs cycle), to be related to the “effort” of nuclear genes OXPHOS to co-evolve and co-adapt with the corresponding, rapidly evolving, mitochondrial genes. The co-adaptation of cooperating genes leads to normal function of the OXPHOS machinery. This coordinated evolution between genomes occur by reciprocal changes in interacting proteins; the deleterious impact of mutations can be “masked” with a mutation in a second site which act as a compensatory mutation [40]. The well-established cooperation between the genomes in separated populations is disrupted when distinct lineages of mtDNA are crossed and the hybrids are less competitive due to low energy production: the distinct mtDNA shows a lack of cooperation with the new nuclear background, affecting mostly the OXPHOS efficiency [12].

The occurrence of 2050 polymorphic sites in 1147 genes, potentially capable of distinguishing between the two lineages may be indicative of a gradual differentiation between “European” and “Anatolian” population with absence of gene flow. The separation of gene pools will, sooner or later, lead to low reproductive success or even to reproductive isolation. The accumulation of non-neutral genetic variation could lead to speciation, with mtDNA polymorphism between populations to drive the differentiation to the associating parts of the nuclear genome, acting as an “engine of speciation”. This hybrid breakdown has been reported in species with distinct geographical populations, such as *Tigriopus californicus*, where crossing of individuals from distinct populations resulted in hybrids with lower energy production [39]. Also, Smith and his colleagues [14] observed impaired reproductive success when crossing brown hares carrying different mitochondrial haplotypes.

To conclude, a plausible scenario to explain the current phylogeographic status of *Lepus europaeus* populations may be the following: (1) According to available molecular data sequence divergence of mitochondrial cytochrome b (Cytb) gene between European and Anatolian lineages ranges from 3.4 to 3.8% (this study, [8]). If we apply the standard calibration of Cytb divergence rate of 2–4% per million years (Myr) [41, 42, 43], with the most possible rate for *Lepus* being 4%, according to fossil data [44] the splitting between the phylogenetic lineages corresponds to 0.85 Myr; (2) During the Late Glacial Maximum (LGM), southern and southeastern European and Anatolian landscapes could have served as refugia for brown hares [45], as indicated to a certain extent by fossil records from the late Pleistocene (see references in [46]; (3) This palaeogeographic situation, under the absence of significant gene flow between European and Anatolian refugia over millennia, has led to differentiated mitochondrial and nuclear gene pools, as indicated by the present and several other studies. For instance sequence divergence for Cytb between closely related *L*. *timidus/L*. *corsicanus* is at 3.3% which is very similar with that between European/Anatolian lineages; (4) At the same time, within isolated populations, mitochondrial and nuclear genes have co-evolved, assuring co-adaptation of cooperating genes and normal function of the cellular energetics; (5) Melting of ice and expansion of populations through various corridors enabled post-glacial colonization of large parts of Europe and Anatolia and has promoted admixtures of populations with different mitochondrial an nuclear genetic backgrounds. The presence of haplotypes of different haplogroups in Bulgaria and North-Eastern Greece indicates a large overlap zone and reveal gene flow from Anatolia to Europe across the late Pleistocene Bosporus land-bridge; (5) In this contact zone it is plausible that the well-established cooperation between the genomes in separated populations is disrupted when distinct lineages of mtDNA are crossed. Distinct mtDNA shows a lack of cooperation with the new nuclear background, affecting mostly the OXPHOS efficiency and the hybrids are less competitive due to low energy production. In this case, this post coupling barrier reinforces reproductive isolation as a first step of an ongoing speciation between these distinct lineages of *L*. *europaeus*.

## Supporting Information

S1 File 9496_hare_cds_nucleotide_seqs.fa. The FASTA file of the 9496 brown hare coding sequences (as nucleotides).
(RAR)

S2 File 9496_hare_cds_protein_seqs.fa. The FASTA file of the 9496 brown hare coding sequences (as proteins).
(FA) Click here for additional data file.

S3 File SNPs.H123full_vs_H456_9496_contigs.vcf. The vcf file generated from Unified Genotyper in GATK that contains information on the identified SNPs and their quality, when comparing the European samples (as a pool) to the Anatolian pooled sample.
(RAR)

S1 Table Blast_human_rabbit.xlsx. An excel file that contains the blast results of the brown hare assembled sequences against human and rabbit (downloaded from ENSEMBL).
(XLSX)

S2 Table Polymorphisms.xlsx. An excel file that contains the following worksheets: **(i)** differentiating_SNPs_2popul: This worksheet contains the 2050 identified polymorphisms that separate the two populations (European vs Anatolian). **(ii)** 69608_polymorphic_sites: This worksheet contains the 69608 identified polymorphic sites found in 7755 out of 9496 brown hare sequences. **(iii)** 7755_genes-cds: This worksheet contains the 7755 brown hare sequences and their annotation (based on Blast) for which we found the 69608 polymorphisms. **(iv)** effect_of_coverage_on_SNP_detec: This worksheet shows the effect of coverage (average number of reads/cds) on the detection of SNPs within a cds. **(v)** Primers_Sanger_validation: This worksheet contains the primer pairs designed for validating with Sanger sequencing a selected subset of RNA-Seq identified SNPs. **(vi)** Sanger validation: This worksheet contains the results from the Sanger validation for a selected set of genes.
(XLSX)
